# The Liver Fluke *Opisthorchis felineus* Exosomal tRNA-Derived Small RNAs as Potential Mediators of Host Manipulation

**DOI:** 10.3390/biom16020244

**Published:** 2026-02-04

**Authors:** Ekaterina Lishai, Maria Pakharukova

**Affiliations:** 1 Institute of Cytology and Genetics, Siberian Branch of the Russian Academy of Sciences, 630090 Novosibirsk, Russia; 2 Department of Natural Sciences, Novosibirsk State University, 630090 Novosibirsk, Russia

**Keywords:** opisthorchiasis, transfer RNA, exosome-like vesicles, tRNA genes, non-coding RNA, RNA interference

## Abstract

The role of extracellular vesicle non-coding RNAs in host–parasite interactions remains poorly understood, particularly for human liver flukes. Although tRNA-derived small RNAs (tsRNAs) are emerging as new regulatory molecules in parasite exosomes, they have not yet been characterized for the liver flukes. We performed small RNA sequencing to profile tsRNAs in the exosome-like vesicles derived from the liver fluke *Opisthorchis felineus*. Transcriptomic data from human cholangiocytes were analyzed to assess the enrichment of the predicted target genes among differentially expressed genes. We identified 247 functional tRNA genes in the *O. felineus* genome. Exosome-like vesicles were highly enriched for particular tsRNAs: derived from tRNA-Asp-GTC, tRNA-Ile-AAT, tRNA-Lys, tRNA-His, and tRNA-Tyr. This enrichment was independent of both genomic tRNA copy number and the amino acid composition of the trematode proteome. In silico prediction revealed that these tsRNAs target human genes involved in cell cycle, migration, and proliferation. Notably, these predicted target genes were significantly enriched among the differentially expressed genes in treated cholangiocytes. Our study provides the first evidence that *O. felineus* exosomes carry a specific repertoire of tsRNAs with the potential to regulate host gene networks. We propose that tsRNAs may contribute to host cell manipulation during *O. felineus* infection.

## 1. Introduction

The liver fluke *Opisthorchis felineus* is a trematode of the Opisthorchiidae family that infests the bile ducts of fish-eating mammals, including humans. Chronic infection with *O. felineus* is a known risk factor for biliary epithelial neoplasia [1,2]. Despite the epidemiological significance of this helminth, the molecular mechanisms underlying the neoplasia development remain poorly understood. Helminths secrete exosome-like vesicles (ELVs) containing non-coding RNAs, proteins, and other biomolecules that are taken up by host cells.

Among the regulatory molecules carried by exosome-like vesicles are tRNA-derived small RNAs (tsRNAs). This diverse class of small non-coding RNAs, generated from mature tRNAs or their precursors, includes tRNA halves (tHFs), tRNA-derived fragments (tRFs), and long tRNA-derived fragments (tLFs) [3]. tsRNAs are found in exosome-like vesicles across diverse species [4,5] and function as regulators in numerous biological and pathological contexts, acting as tumor suppressors, oncogenes, and modulators of protein synthesis, apoptosis, and neurodegeneration [6]. They are broadly categorized by their biogenesis and structure: tRNA halves (28–36 nucleotides) are produced in response to cellular stress (e.g., nutrient deprivation, oxidative stress) primarily through cleavage by angiogenin, yielding 5′- and 3′-halves [7,8]. In contrast, tRNA fragments result from Dicer-dependent or independent cleavage within the D- or T-loops [9], while long tRNA fragments are generated by cleavage adjacent to these loops [3].

Notably, tsRNAs have been identified in the exosome-like vesicles of numerous parasites and are hypothesized to mediate host gene regulation [10,11,12,13,14]. Their proposed mechanism mirrors that of microRNAs; they can bind to complementary sequences in target host mRNAs, leading to transcript degradation or translational repression, or they can interact with RNA-binding proteins to influence mRNA stability [15].

Given the critical gap in understanding how the liver flukes influences host cells, this study aimed to characterize the tsRNA repertoire within *O. felineus* exosome-like vesicles and investigate their potential regulatory functions. We combined small RNA sequencing of parasite vesicles with in silico prediction of tsRNA targets in the human genome and integrated these predictions with transcriptomic data from treated human cholangiocytes. Our findings provide first insights into mechanisms of host–parasite interaction, suggesting that vesicular tsRNAs may contribute to the pathogenic outcomes of liver fluke infection.

## 2. Materials and Methods

### 2.1. Animals

Two-month-old golden hamsters (*Mesocricetus auratus*) from the Animal Facility of the ICG SB RAS were used in the study. *O. felineus* metacercariae were isolated from freshwater fish (*Leuciscus idus*) caught in the Ob River near Novosibirsk. The hamsters were orally infected with 75 metacercariae each. Adult trematodes were removed from the hamsters’ bile ducts two months after the infection and washed 10 times with sterile saline [16]. The animal study protocol was approved by the Ethics Committee of the Institute of Cytology and Genetics, Siberian Branch of the Russian Academy of Sciences (protocol code 42 of 25 May 2018).

### 2.2. Isolation of Exosome-like Vesicles

Adult trematodes were maintained in the medium (RPMI 1640 medium, 1% glucose, 100 units/mL penicillin G, 100 μg/mL streptomycin and 0.25 μg/mL amphotericin B), then exosome-like vesicles were isolated from the medium as described previously [17]. Transmission electron microscopy of EVs derived from *O. felineus* is presented in Figure S1.

### 2.3. Small RNAs Extraction, Library Preparation and Sequencing

Small RNAs were extracted from exosome-like vesicles with the miRNeasy Serum/Plasma Kit (Qiagen, Shanghai, China). Libraries for sequencing were prepared using the MGIEasy Small RNA Library Prep Kit V2.0 (MGI Tech., Shenzhen, China).

The three DNA libraries were subjected to single-end (1 × 50) sequencing on the BGISEQ-500 platform (BGI Genomics, Hong Kong, China). Sequencing data quality was checked using FastQC (v.0.11.9). Basic sequencing statistics are presented in Table S1.

### 2.4. Prediction of the O. felineus tRNA

The amino acid composition of *O. felineus* proteins was determined based on the predicted trematode protein sequences (GCA_004794785.1).

For tRNA prediction, the *O. felineus* genome (GCA_004794785.1) was used, along with tRNAscan-SE 2.0 v.2.0.9 with the following parameters: Search Mode = Eukaryotic, Isotype-specific model scan = yes, Infernal first pass cutoff score = 10 [18]. Of the 4523 predicted sequences, pseudogenes (4276) were removed. The remaining sequences (247), identified as genes, were used for further analysis (Table S2).

### 2.5. Search for tsRNAs in Small Noncoding RNAs from O. felineus Exosome-like Vesicles

Small RNA libraries derived from *O. felineus* exosome-like vesicles were aligned to predicted tRNA sequences using STAR v.2.7.10b [19] (Table S3). Initial mapping revealed that many predicted tRNA sequences corresponded to different isoforms of the same tRNA, distinguished by a small number of nucleotide substitutions, insertions, or deletions. This sequence similarity caused a significant proportion of reads to map to multiple loci, precluding reliable quantitative analysis.

To resolve this, we selected a single, representative sequence for each tRNA isoacceptor molecule. This procedure yielded a non-redundant set of 50 tRNA sequences. The small RNAs were then re-mapped to this refined reference set using STAR. Read counts mapping to each tRNA sequence were quantified using samtools coverage v.1.22, (https://www.htslib.org/doc/samtools-coverage.html [accessed on 30 January 2026]). The results are presented in Table S1.

The dominant types of tRNA-derived small RNAs (tsRNAs) were classified based on their length and cleavage position relative to the canonical tRNA structure using MEGA v.11.0.13 (https://www.megasoftware.net/ [accessed on 30 January 2026]). Data visualization was performed using LibreOffice Calc v.24.2.7.2 (https://www.libreoffice.org/discover/calc/ [accessed on 30 January 2026]), and figures were assembled in Inkscape v.1.4.2 (https://inkscape.org/ru/ [accessed on 30 January 2026]).

### 2.6. Functional Analysis of tsRNAs from O. felineus Exosome-like Vesicles

To identify potential target genes of dominant tsRNAs in the human genome, we performed an in silico analysis using tRFtarget 2.0 [20]. It utilizes the RNAhybrid and IntaRNA algorithms to predict binding sites and then assess where their predictions overlap to find reliable binding sites based on the consensus of the two predictions. We selected for this analysis tsRNAs that represented more than 2% of all mapped reads from the trematode exosome-like vesicles. The target sequences comprised the 3′-untranslated regions (3′-UTRs) of human genes involved in key pathways, including cell cycle (MsigDB: M7963), cell migration (MsigDB: M2001), proliferation (GO:0008283), and the negative regulation of these processes (cell cycle: GO:M12829; migration: GO:0030336; proliferation: GO:M6566; apoptotic process: GO:0043066). 3′-UTR sequences were retrieved from the Ensembl Biomart database (Ensembl Genes 115, GRCh38.p14). The list of genes is presented in Table S4. An interaction between a tsRNA and a gene was considered significant if the predicted binding energy was below −15 kcal/mol for both algorithms.

### 2.7. Transcriptome Data Analysis

To validate the biological relevance of the target gene prediction, we analyzed the transcriptome data from human H69 cholangiocytes treated with *O. felineus* excretory-secretory product (NCBI BioProject (PRJNA1203356: ESP treated samples—SAMN45991479, SAMN45991480, SAMN45991481; untreated—SAMN45991473, SAMN45991474, SAMN45991475). Quality of the raw data was assessed using FastQC v.0.11.9. The reads were mapped to the human GRCh38 genome using STAR. Analysis of differentially expressed genes (DEGs) was conducted using the DESeq2 v.1.42.0 R package [21] (Wald test with a threshold of 0.05), and genes with an adjusted *p*-value (P_adj_ < 0.05) were defined as DEGs. The data on the transcriptome analysis are presented in Table S5. PCA plot showing transcriptome differences between untreated and ESP-treated H69 cholangiocytes is presented in Figure S2. A heatmap for DEGs in H69 cholangiocytes genes (P_adj_ < 0.05) is presented in Figure S3 (color corresponds to per-gene z-score).

The statistical significance of the enrichment of differentially expressed genes among the predicted tsRNA targets was assessed using a Pearson’s chi-square test with Yates’s correction (stats v.4.3.3 R package). The list of target genes for transcription factor c-FOS was performed from database Harmonizome 3.0 (https://maayanlab.cloud/Harmonizome/ [accessed on 30 January 2026]).

Visualization of the relationships between tsRNAs and their target genes was created using the SankeyMATIC web service (https://sankeymatic.com/ [accessed on 30 January 2026]).

## 3. Results

### 3.1. Identification of tRNA Genes and Description of the Amino Acid Composition of Proteins Based on the O. felineus Genome Data

**Figure 1:**
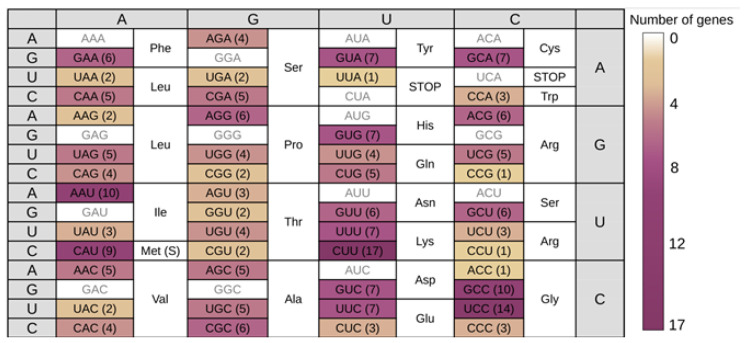
The distribution of genes for each isoacceptor tRNAs in *O. felineus* genome.

We identified 4523 putative tRNA genes within the *O. felineus* genome. Based on primary sequence and secondary structure analysis, 4276 of these were classified as pseudogenes [20], leaving 247 confirmed tRNA genes. For these functional tRNAs, the anticodon and cognate amino acid were determined. The distribution of genes for each isoacceptor tRNA is shown in Figure 1.

**Figure 2:**
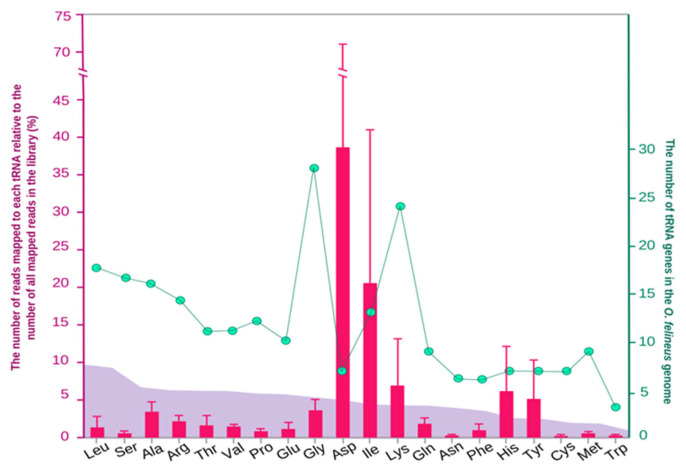
Comparative analysis of tsRNA abundance, tRNA gene number in the genome, and proteomic amino acid composition in *O. felineus*. Pink bars: The relative abundance of small RNA reads mapping to tRNAs for each amino acid (summed across all isoacceptors); Turquoise dots: The number of tRNA genes encoding each amino acid in the genome; Purple zone: The percentage of each amino acid in the *O. felineus* proteome. Libraries from exosomes are enriched for tsRNAs derived from Asp, Ile, Lys, His, and Tyr tRNAs.

Analysis of 21,413 *O. felineus* protein sequences revealed their amino acid composition (Figure 2). The most prevalent amino acid residues were leucine (9.87%) and serine (9.45%), in contrast to the least prevalent, methionine (2.02%) and tryptophan (1.13%).

### 3.2. Small RNA Libraries from O. felineus Exosome-like Vesicles Are Enriched in Fragments and Halves of tRNAs of the Aspartic Acid, Isoleucine, Lysine, Histidine, and Tyrosine

Initial mapping of small RNA libraries from *O. felineus* exosome-like vesicles to the predicted tRNA genes revealed that reads mapped redundantly to multiple genomic copies of the same tRNA. This indicates that the trematode genome contains numerous near-identical tRNA gene duplicates, differing by minor polymorphisms. Due to this multi-mapping, the number of uniquely mapped reads was insufficient for reliable quantification, as they primarily represented these polymorphic sites.

To enable a quantitative analysis, we curated a non-redundant reference set by selecting the most abundant tRNA sequence for each anticodon variant, based on the initial read mappings. The small RNA libraries were then realigned to this curated set. This analysis revealed that the dominant tsRNAs were derived from tRNA-Asp (38.66% of all mapped reads), tRNA-Ile (20.56%), tRNA-Lys (6.93%), tRNA-His (6.18%), and tRNA-Tyr (5.15%) (Figure 2).

The pronounced abundance of these specific tsRNAs prompted us to investigate its basis. We hypothesized that their enrichment in exosome-like vesicles could be driven by: (i) the frequency of their cognate amino acids in the *O. felineus* proteome, (ii) the genomic copy number of the corresponding tRNA genes, or (iii) their intrinsic molecular stability. To test these hypotheses, we compared the relative abundance of tsRNAs with the amino acid composition of the trematode proteome and the number of tRNA genes for each amino acid. We found no correlation, ruling out the first two factors as primary drivers of the observed tsRNA enrichment in EVs (Figure 2).

### 3.3. Most tsRNAs from O. felineus Exosome-like Vesicles Are 5′-tRF and 5′-tHF

**Figure 3:**
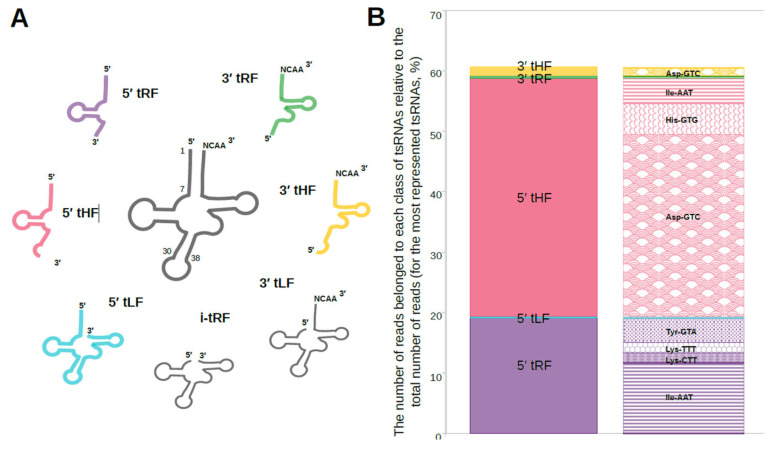
Characterization of tsRNA fragments from *O. felineus* exosome-like vesicles. (**A**) Classification of the dominant tsRNA types derived from the most abundant tRNAs. (**B**) Relative abundance of each tsRNA type. The tsRNAs in (**A**,**B**) have the same color to demonstrate the relationship between the fragment structure and abundance.

To characterize the diversity of tsRNA types, we analyzed the most abundant tsRNAs derived from the dominant isoacceptor tRNAs: tRNA-Asp-GTC, tRNA-His-GTG, tRNA-Ile-AAT, tRNA-Ile-TAT, tRNA-Lys-CTT, tRNA-Lys-TTT, and tRNA-Tyr-GTA. The most prevalent fragments from these tRNAs, which collectively accounted for >60% of all mapped reads, were classified (Figure 3A).

The 5′-tHF type was the most abundant, representing 39.41% of classified fragments and originating primarily from tRNA-Asp-GTC, tRNA-His-GTG, and tRNA-Ile-AAT. The 5′-tRF type accounted for approximately 19% of fragments, derived from tRNA-Ile-AAT, tRNA-Lys-CTT, tRNA-Lys-TTT, and tRNA-Tyr-GTA. In contrast, 3′-end fragments were less common, with 3′-tHF (1.36%) originating from tRNA-Asp-GTC and 3′-tRF (0.48%) and 5′-tLF (0.29%) being the least represented types (Figure 3B).

### 3.4. Functional Characteristics of Major tsRNAs

**Figure 4:**
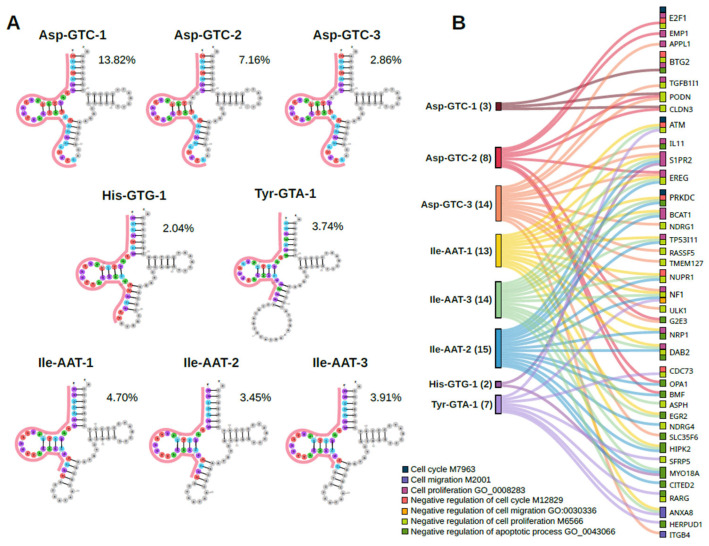
Identification of tsRNA target genes among differentially expressed genes in *O. felineus* excretory-secretory product-treated human cholangiocytes. (**A**) Abundant *O. felineus* tsRNAs selected for target prediction in the human genome. (**B**) Sankey diagram linking these tsRNAs to their predicted target genes that were differentially expressed in H69 cholangiocytes following treatment with the trematode excretory-secretory product.

To predict the regulatory functions of *O. felineus* tsRNAs in human cells, we selected tsRNAs that were highly enriched in exosome-like vesicles, each constituting at least 2% of all mapped reads (Figure 4A). Since tsRNAs do not have generally accepted names, for convenience we designated them according to the scheme “Amino acid”–“Anticodon”–“fragment number by abundance.” Thus, Asp-GTC-1 represents the most abundant fragment of aspartic acid tRNA with the GTC anticodon. To search for tsRNA target genes, we analyzed the 3′UTRs of genes involved in the regulation of the cell cycle, migration, proliferation, and their negative regulation, as well as those participating in the negative regulation of apoptosis, since these processes were previously clearly observed on human cholangiocytes treated with *O. felineus* ESP [22].

The number of predicted target genes for each cellular pathway is presented in Table 1. The highest number of target genes was identified for tsRNA 5′-tHF-Asp-GTC-3 (206 genes), and the lowest for tsRNA 5′-tHF-His-GTG-1 (34 genes).

Given that tRNA fragments likely interact with target mRNAs through a mechanism analogous to microRNA-mediated RNA interference [23], we investigated whether their predicted target genes were differentially expressed in human cholangiocytes treated with the *O. felineus* excretory-secretory product (which contains exosome-like vesicles [24]). We observed a significant enrichment of differentially expressed genes among the predicted tsRNA targets (Pearson’s Chi-squared test with Yates’ continuity correction = 26.573, df = 1, *p*-value = 2.538 × 10^−7^). Specifically, 38 out of 1301 differentially expressed genes were identified as tsRNA targets (Figure 4B).

Furthermore, we found that some genes were targeted by multiple tsRNAs simultaneously. The *EREG*, *NF1*, and *S1PR2* genes were targeted by five different tRNA fragments. In contrast, 19 target genes were unique to a single tsRNA. For the tsRNA Asp-GTC-3, nine genes (*IL11*, *SLC35F6*, *NDRG1*, *RASSF5*, *TGFB1l1*, *TMEM127*, *ULK1*, *APPL1*, and *ITGB4*) fell into this category (Table S6).

## 4. Discussion

Small tRNA-derived RNAs (tsRNAs) are an emerging class of regulatory noncoding RNAs studied as disease biomarkers and signaling molecules. Some studies revealed that tsRNAs are known components of parasitic exosomes [3,14,25], their role in host–parasite interactions remains largely unexplored. Our study provides the prediction of tRNA genes in the trematode *Opisthorchis felineus* and an analysis of tsRNAs derived from parasitic exosome-like vesicles.

Our analysis revealed that *O. felineus* genome contains multiple copies of tRNA genes, often organized in clusters [26], which is in line with many other eukaryotic species. However, the genomic architecture of these loci requires further investigation.

Strikingly, the abundance of tsRNAs in exosome-like vesicles did not reflect the genomic copy number of their corresponding tRNA genes nor the amino acid composition of the *O. felineus* proteome. Instead, we observed an enrichment of tsRNAs derived from aspartic acid, isoleucine, histidine, tyrosine, and lysine tRNAs.

Unfortunately, no data are available on the selective packaging of tsRNAs into exosome-like vesicles. In contrast, for microRNAs the roles for some proteins for such selective packaging were revealed. In particular, some RNA-binding proteins such as Y-box-binding protein 1 (YBX1), heterogeneous nuclear ribonucleoprotein A2/B1 (hnRNP A2/B1), and synaptotagmin-binding cytoplasmic RNA-interacting protein (SYNCRIP) are involved in their selective loading into vesicles. The interaction of these proteins with RNA requires the presence of specific short sequence motifs in the RNA molecule [27]. Various post-translational modifications, such as SUMOylation or phosphorylation, also are important in the selective shuttle of microRNAs into EVs. Notably, the endoplasmic reticulum is also implicated in the formation of RNA-enriched exosomes [28].

This enrichment suggests a functional, non-stochastic role for these particular tsRNAs, probably involved in RNA interference or other regulatory processes within the host. This hypothesis is supported by some data on regulatory functions of tsRNAs in other systems. For instance, human tsRNA-Asp-GTC induce inflammatory response via the tRF-Asp-GTC/galectin-3/TLR4/NF-κB pathway in vascular cells [29], while another tsRNA suppresses tumor growth by displacing oncogenic transcripts from YBX1 under hypoxia stress [30]. Notably, tsRNA-Asp-GTC is also highly abundant in *Entamoeba histolytica* exosomes, is capable of binding to Argonaute proteins, and is stress-inducible [31], in line with our findings of Asp-GTC enrichment. Furthermore, a tsRNA-Ile-AAT has been shown to downregulate *SERPINE1* expression in human keratinocytes [32]. These parallels strengthen the possibility that the abundant *O. felineus* tsRNAs are functionally active in host cell manipulation.

The enrichment of particular tsRNAs in *O. felineus* exosome-like vesicles suggests a targeted packaging mechanism beyond passive accumulation. While actors such as intrinsic tsRNA stability or stress-induced fragmentation could contribute, a comparison with other trematodes argues against a universal explanation. For instance, the most abundant tsRNAs in exosomes from another liver fluke *Fasciola hepatica* (5′-tHF-Gly-GCC, 5′-tLF-Gln-CTG, and 5′-tHF-Cys-GCA) [3] are distinct from those we identified in *O. felineus*. This species-specific tsRNA signature indicates that abundance is not dictated solely by general stability or stress responses but likely reflects functional specialization unique to each parasite. Nevertheless, the predominance of 5′ tsRNAs may be associated with the greater stability of these fragments compared with 3′ tsRNAs. In silico analysis has shown that 5′ halves derived from tRNA-Gly and tRNA-Glu can form homo- and heterodimers, whereas spontaneous dimerization of 5′ halves from tRNA-Gly has been observed in vitro. The formation of such structures protects 5′ tsRNAs from RNase degradation [33]. Moreover, some 5′ tsRNAs (stress-induced 5′-tsRNA-Ala and 5′-tsRNA-Cys) are capable of forming G-quadruplex (G4) structures [34], which are known to be highly stable.

This hypothesis about functional activity of tsRNAs is supported by our findings in human cholangiocytes. Treatment with the *O. felineus* excretory-secretory product (containing exosome-like vesicles) is known to alter the cell proliferation and migration rates [22]. We found that the predicted target genes of the tsRNAs are significantly overrepresented among the differentially expressed genes in these treated human cells. This suggests that exosomal tsRNAs are capable of modulation of these host cellular processes during infection. The observed upregulation of some target genes could be explained by non-canonical mechanisms of gene regulation, as previously documented for microRNAs [35].

Among the predicted target genes of tsRNAs, the NF1 gene deserves special attention. It is a target for five of the most abundant OF tsRNAs, and its expression level in human cholangiocytes was significantly decreased after ESP treatment. After the cellular uptake as a cargo of exosome-like vesicles via clathrin-dependent endocytosis [17], tsRNAs may bind to the NF1 mRNA, leading to the silencing of the tumor suppressor NF1 protein output, thereby activating the Ras signaling pathway [36]. The Ras pathway strongly activates the c-FOS gene, a key player in cell differentiation and proliferation. Notably, we observed c-FOS among DEGs in human cholangiocytes treated with ESP (Padj < 0.05; Log_2_FC > 0.6). c-FOS transcription factor regulates over 13,000 target genes according to the Harmonizome 3.0 database. Among the 1301 DEGs in cholangiocytes (Padj < 0.05), the mRNA expression level of 82 c-FOS known target genes was upregulated (Log_2_FC > 0.6) (Table S5). These genes are primarily associated with apoptosis and cell proliferation pathways.

Finally, we acknowledge that the host cell phenotype is likely the result of a synergistic effect from the complex mixture of biomolecules in the excretory-secretory product and exosomes [18]. The contribution of other regulatory molecules, such as microRNAs, which are known to be functional in the exosomes of other trematodes like *F. hepatica*, *O. viverrini*, and *Clonorchis sinensis* [3,37,38,39,40,41], requires further investigation. Furthermore, given evidence that exosomes mediate inter-species communication [29,42], the potential autocrine or paracrine effects of tsRNAs on the trematodes themselves represent an interesting new way for future research.

## 5. Conclusions

Overall, the mechanisms by which the liver flukes contribute to the development of biliary neoplasia, including the role of parasite-derived exosomes, remain poorly defined. This study provides the first characterization of tRNA-derived small RNAs (tsRNAs) in *O. felineus* exosome-like vesicles. We also identified their potential target genes in the human genome. These tsRNAs may function as host interfering regulatory molecules capable of modulating host gene expression. Unfortunately, in silico analysis alone does not allow us to establish a definitive causal relationship between the presence of specific tsRNAs in exosome-like vesicles and the altered expression of their predicted target genes in recipient cells. Another limitation is that the pathway “Negative regulation of cell migration” has only 15 genes, which may limit statistical robustness.

Nevertheless, to move from prediction to established mechanism, the functionality of these tsRNAs needs further validation. Future research is needed to employ molecular and cellular approaches to confirm the activity, such as (i) assessing cell proliferation and migration rates of human cells treated with tsRNA mimics or inhibitors; and (ii) validating target interactions using reporter constructs containing the 3′-uncoded regions of mRNA target genes. Thus, further experiments are needed to elucidate the contribution of trematode tsRNAs to the host–parasite interaction and their potential role in biological carcinogenesis.

## Figures and Tables

**Table 1:** Prediction of tsRNA target genes associated with host regulatory pathways.

Pathway	Total Gene Number in the Pathway	Target Gene Number	Percentage of Genes in a Pathway That Are Target Genes
Cell cycle M7963	125	27	21.6%
Cell migration M2001	184	26	14.1%
Cell proliferation GO_0008283	512	98	19.1%
Negative regulation of cell cycle M12829	398	71	17.8%
Negative regulation of cell migration GO:0030336	15	7	46.6%
Negative regulation of cell proliferation M6566	792	124	15.7%
Negative regulation of apoptotic process GO:004306	786	137	17.4%

## Data Availability

All data are included in the manuscript and/or Supplemental Materials.

## Supplementary Materials

The following supporting information can be downloaded at https://www.mdpi.com/article/10.3390/biom16020244/s1, Table S1: Small RNA libraries basic statistics; Table S2: Sequences of predicted tRNA genes of the *O. felineus* (FASTA-files); Table S3: Raw sequences of mapped reads to the tRNA gene sequences (FASTA-files in ZIP archive); Table S4: List of genes and 3′UTR transcripts used for target gene prediction; Table S5: H69 NGS library basic statistics and gene expression analysis; Table S6: Target genes for major tsRNAs and their mRNA expression levels (Log_2_FC and Padj) in H69 cholangiocytes after *O. felineus* ESP treatment; Figure S1: Transmission electron microscopy of exosome-like vesicles derived from *O. felineus* (A, B, C—three biological replicates); Figure S2: PCA plot showing transcriptome differences between untreated and ESP-treated H69 cholangiocytes; Figure S3: A heatmap for differentially expressed genes in H69 cholangiocytes (color corresponds to per-gene z-score).

## Abbreviations

The following abbreviations are used in this manuscript:

tRNA	Transfer RNA
tsRNAs	tRNA-derived small RNAs
mRNA	Messenger RNA
ESP	Excretory-secretory product
5′-HF/3′-HF	tRNA halves
5′-TF/3′-TF	tRNA fragments
5′-LF/3′-LF	long tRNA fragments
ELVs	Exosome-like vesicles
Asp	Aspartic acid
Ile	Isoleucine
Lys	Lysine
His	Histidine
Tyr	Tyrosine
Gly	Glycine
Gln	Glutamine
Cys	Cysteine

## References

[1] Xia J., Jiang S.C., Peng H.J. (2015). Association between Liver Fluke Infection and Hepatobiliary Pathological Changes: A Systematic Review and Meta-Analysis. PLoS ONE.

[2] Lishai E.A., Zaparina O.G., Kapushchak Y.K., Sripa B., Hong S.J., Cheng G., Pakharukova M.Y. (2024). Comparative liver transcriptome analysis in hamsters infected with food-borne trematodes *Opisthorchis felineus*, *Opisthorchis viverrini*, or *Clonorchis sinensis*. PLoS Negl. Trop. Dis..

[3] Fontenla S., Langleib M., de la Torre-Escudero E., Domínguez M.F., Robinson M.W., Tort J. (2022). Role of *Fasciola hepatica* Small RNAs in the Interaction With the Mammalian Host. Front. Cell. Infect. Microbiol..

[4] Zhao F., Cheng L., Shao Q., Chen Z., Lv X., Li J., He L., Sun Y., Ji Q., Lu P. (2020). Characterization of serum small extracellular vesicles and their small RNA contents across humans, rats, and mice. Sci. Rep..

[5] Zhou Y., Tao D., Shao Z., Wang X., Xu J., Li Y., Li K. (2022). Expression profiles of exosomal tRNA-derived fragments and their biological functions in lipomas. Front. Cell Dev. Biol..

[6] Hou J., Li Q., Wang J., Lu W. (2022). tRFs and tRNA Halves: Novel Cellular Defenders in Multiple Biological Processes. Curr. Issues Mol. Biol..

[7] Li G., Das S. (2025). Self-quenched tRNA reporters for imaging tRNA-derived RNA biogenesis. Methods Enzymol..

[8] Jin H., Yeom J.H., Shin E., Ha Y., Liu H., Kim D., Joo M., Kim Y.H., Kim H.K., Ryu M. (2024). 5′-tRNAGly(GCC) halves generated by IRE1α are linked to the ER stress response. Nat. Commun..

[9] Berg M.D., Brandl C.J. (2021). Transfer RNAs: Diversity in form and function. RNA Biol..

[10] Fernandez-Calero T., Garcia-Silva R., Pena A., Robello C., Persson H., Rovira C., Naya H., Cayota A. (2015). Profiling of small RNA cargo of extracellular vesicles shed by *Trypanosoma cruzi* reveals a specific extracellular signature. Mol. Biochem. Parasitol..

[11] Lambertz U., Oviedo Ovando M.E., Vasconcelos E.J., Unrau P.J., Myler P.J., Reiner N.E. (2015). Small RNAs derived from tRNAs and rRNAs are highly enriched in exosomes from both old and new world *Leishmania* providing evidence for conserved exosomal RNA Packaging. BMC Genom..

[12] Nowacki F.C., Swain M.T., Klychnikov O.I., Niazi U., Ivens A., Quintana J.F., Hensbergen P.J., Hokke C.H., Buck A.H., Hoffmann K.F. (2015). Protein and small non-coding RNA-enriched extracellular vesicles are released by the pathogenic blood fluke *Schistosoma mansoni*. J. Extracell. Vesicles.

[13] Artuyants A., Campos T.L., Rai A.K., Johnson P.J., Dauros-Singorenko P., Phillips A., Simoes-Barbosa A. (2020). Extracellular vesicles produced by the protozoan parasite *Trichomonas vaginalis* contain a preferential cargo of tRNA-derived small RNAs. Int. J. Parasitol..

[14] Natali L., Luna Pizarro G., Moyano S., de la Cruz-Thea B., Musso J., Rópolo A.S., Eichner N., Meister G., Musri M.M., Feliziani C. (2023). The exosome-like vesicles of *Giardia* assemblages A, B, and E are involved in the delivering of distinct small RNA from parasite to parasite. Int. J. Mol. Sci..

[15] Yu X., Xie Y., Zhang S., Song X., Xiao B., Yan Z. (2021). tRNA-derived fragments: Mechanisms underlying their regulation of gene expression and potential applications as therapeutic targets in cancers and virus infections. Theranostics.

[16] Pakharukova M.Y., Pakharukov Y.V., Mordvinov V.A. (2018). Effects of miconazole/clotrimazole and praziquantel combinations against the liver fluke *Opisthorchis felineus* in vivo and in vitro. Parasitol. Res..

[17] Pakharukova M.Y., Savina E., Ponomarev D.V., Gubanova N.V., Zaparina O., Zakirova E.G., Cheng G., Tikhonova O.V., Mordvinov V.A. (2023). Proteomic characterization of *Opisthorchis felineus* exosome-like vesicles and their uptake by human cholangiocytes. J. Proteom..

[18] Chan P.P., Lin B.Y., Mak A.J., Lowe T.M. (2021). tRNAscan-SE 2.0: Improved detection and functional classification of transfer RNA genes. Nucleic Acids Res..

[19] Dobin A., Davis C.A., Schlesinger F., Drenkow J., Zaleski C., Jha S., Batut P., Chaisson M., Gingeras T.R. (2013). STAR: Ultrafast universal RNA-seq aligner. Bioinformatics.

[20] Li N., Yao S., Yu G., Lu L., Wang Z. (2024). tRFtarget 2.0: Expanding the targetome landscape of transfer RNA-derived fragments. Nucleic Acids Res..

[21] Love M.I., Huber W., Anders S. (2014). Moderated estimation of fold change and dispersion for RNA-seq data with DESeq2. Genome Biol..

[22] Ponomarev D., Zaparina O., Kovner A., Hadieva E., Persidskij M., Pakharukova M. (2025). The EGFR signaling pathway is involved in the biliary intraepithelial neoplasia associated with liver fluke infection. Pathogens.

[23] Gong M., Deng Y., Xiang Y., Ye D. (2023). The role and mechanism of action of tRNA-derived fragments in the diagnosis and treatment of malignant tumors. Cell Commun. Signal..

[24] Ponomarev D.V., Lishai E.A., Kovner A.V., Kharkova M.V., Zaparina O., Kapuschak Y.K., Mordvinov V.A., Pakharukova M.Y. (2023). Extracellular vesicles of the liver fluke *Opisthorchis felineus* stimulate the angiogenesis of human umbilical vein endothelial cells. Curr. Res. Parasitol. Vector-Borne Dis..

[25] Kusakisako K., Nakao R., Katakura K. (2023). Detection of parasite-derived tRNA and rRNA fragments in the peripheral blood of mice experimentally infected with *Leishmania donovani* and *Leishmania amazonensis* using next-generation sequencing analysis. Parasitol. Int..

[26] Bermudez-Santana C., Attolini C.S., Kirsten T., Engelhardt J., Prohaska S.J., Steigele S., Stadler P.F. (2010). Genomic organization of eukaryotic tRNAs. BMC Genom..

[27] Abdelgawad A., Huang Y., Gololobova O., Yu Y., Witwer K.W., Parashar V., Batish M. (2025). Defining the Parameters for Sorting of RNA Cargo Into Extracellular Vesicles. J. Extracell. Vesicles.

[28] Lee Y.J., Shin K.J., Chae Y.C. (2024). Regulation of cargo selection in exosome biogenesis and its biomedical applications in cancer. Exp. Mol. Med..

[29] Wang C., Yu B., Zhou H., Li H., Li S., Li X., Wang W., Feng Y., Yu T. (2025). tRF-AspGTC promotes intracranial aneurysm formation by controlling TRIM29-mediated galectin-3 ubiquitination. Research.

[30] Goodarzi H., Liu X., Nguyen H.C., Zhang S., Fish L., Tavazoie S.F. (2015). Endogenous tRNA-derived fragments suppress breast cancer progression via YBX1 displacement. Cell.

[31] Sharma M., Morgado P., Zhang H., Ehrenkaufer G., Manna D., Singh U. (2020). Characterization of extracellular vesicles from *Entamoeba histolytica* identifies roles in intercellular communication that regulates parasite growth and development. Infect. Immun..

[32] Zeng J., Xie Y., Zhang H., Zhang Y., Zhang Y., Liu L., Hu Q., Zhou L., Gao L., Tan W. (2023). Protective roles of tRNA-derived small RNA tRF-Ile-AAT-019 in pathological progression of psoriasis. Exp. Dermatol..

[33] Tosar J.P., Gámbaro F., Darré L., Pantano S., Westhof E., Cayota A. (2018). Dimerization confers increased stability to nucleases in 5′ halves from glycine and glutamic acid tRNAs. Nucleic Acids Res..

[34] Ivanov P., O’Day E., Emara M.M., Wagner G., Lieberman J., Anderson P. (2014). G-quadruplex structures contribute to the neuroprotective effects of angiogenin-induced tRNA fragments. Proc. Natl. Acad. Sci. USA.

[35] Valinezhad Orang A., Safaralizadeh R., Kazemzadeh-Bavili M. (2014). Mechanisms of miRNA-mediated gene regulation from common downregulation to mRNA-specific upregulation. Int. J. Genom..

[36] Hilal N., Chen Z., Chen M.H., Choudhury S. (2023). RASopathies and cardiac manifestations. Front. Cardiovasc. Med..

[37] Tran N., Ricafrente A., To J., Lund M., Marques T.M., Gama-Carvalho M., Cwiklinski K., Dalton J.P., Donnelly S. (2021). *Fasciola hepatica* hijacks host macrophage miRNA machinery to modulate early innate immune responses. Sci. Rep..

[38] Chaiyadet S., Sotillo J., Smout M., Cooper M., Doolan D.L., Waardenberg A., Eichenberger R.M., Field M., Brindley P.J., Laha T. (2023). Small extracellular vesicles but not microvesicles from *Opisthorchis viverrini* promote cell proliferation in human cholangiocytes. bioRxiv.

[39] Wen L., Li M., Yin J. (2024). PTEN deficiency induced by extracellular vesicle miRNAs from *Clonorchis sinensis* potentiates cholangiocarcinoma development by inhibiting ferroptosis. Int. J. Mol. Sci..

[40] Zhang B., Li X., Zhou Q.Y., Zhang C., Bian Z.R., Ren X.X., Yu Q., Hua H., Jiang Z., Zhang B. (2025). *Clonorchis sinensis* extracellular vesicles associated with Csi-let-7a-5p activate pro-inflammatory macrophages to induce biliary injury. PLoS Negl. Trop. Dis..

[41] Wu Y., Yi X., Li M., Xu A., Wu A., Zhong Z., Li X. (2025). Effect and mechanism of Csi-miR-125a induced liver fibrosis in the exosomes of *Clonorchis sinensis*. Chin. J. Parasitol. Parasit. Dis..

[42] Salas N., Blasco Pedreros M., Dos Santos Melo T., Maguire V.G., Sha J., Wohlschlegel J.A., Pereira-Neves A., de Miguel N. (2023). Role of cytoneme structures and extracellular vesicles in *Trichomonas vaginalis* parasite-parasite communication. eLife.

