# Supplementary figures and images for: The Liver Fluke Opisthorchis felineus Exosomal tRNA-Derived Small RNAs as Potential Mediators of Host Manipulation
Source: Biomolecules. 2026 Feb 4;16(2):244. doi: 10.3390/biom16020244 (PMC12938311; doi:10.3390/biom16020244)

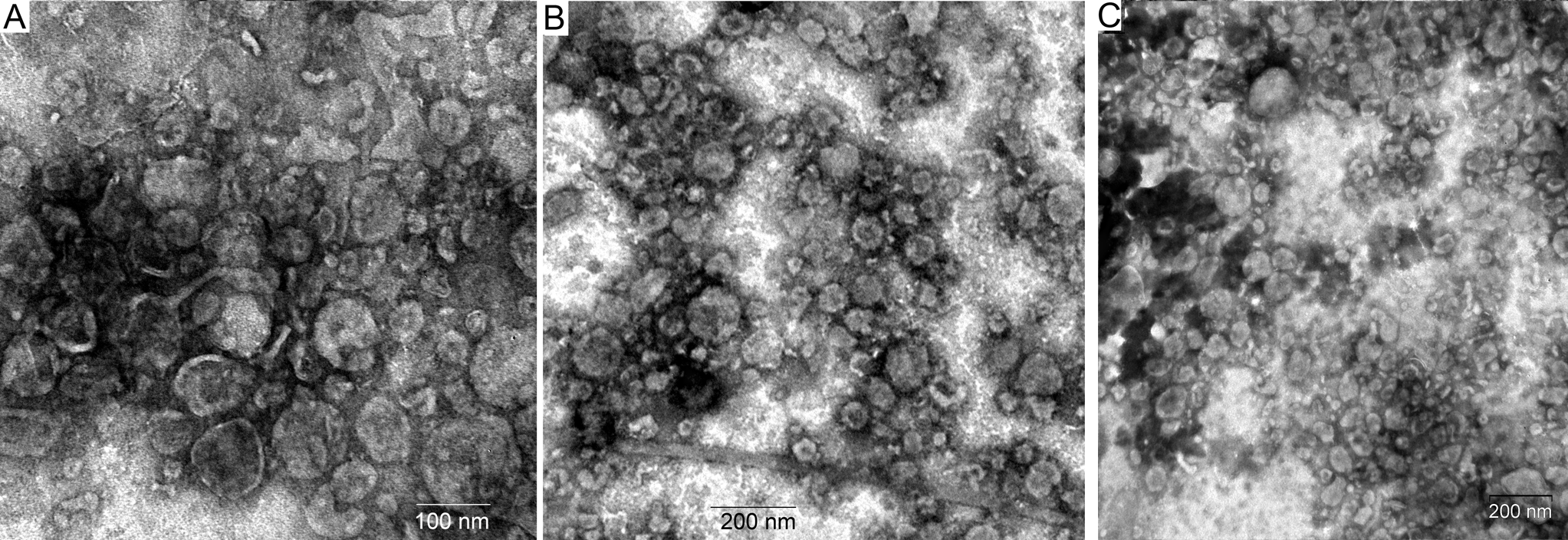

Supplement: Supplementary file 1 [file biomolecules-16-00244-s001.zip › Figure_S1.tif]

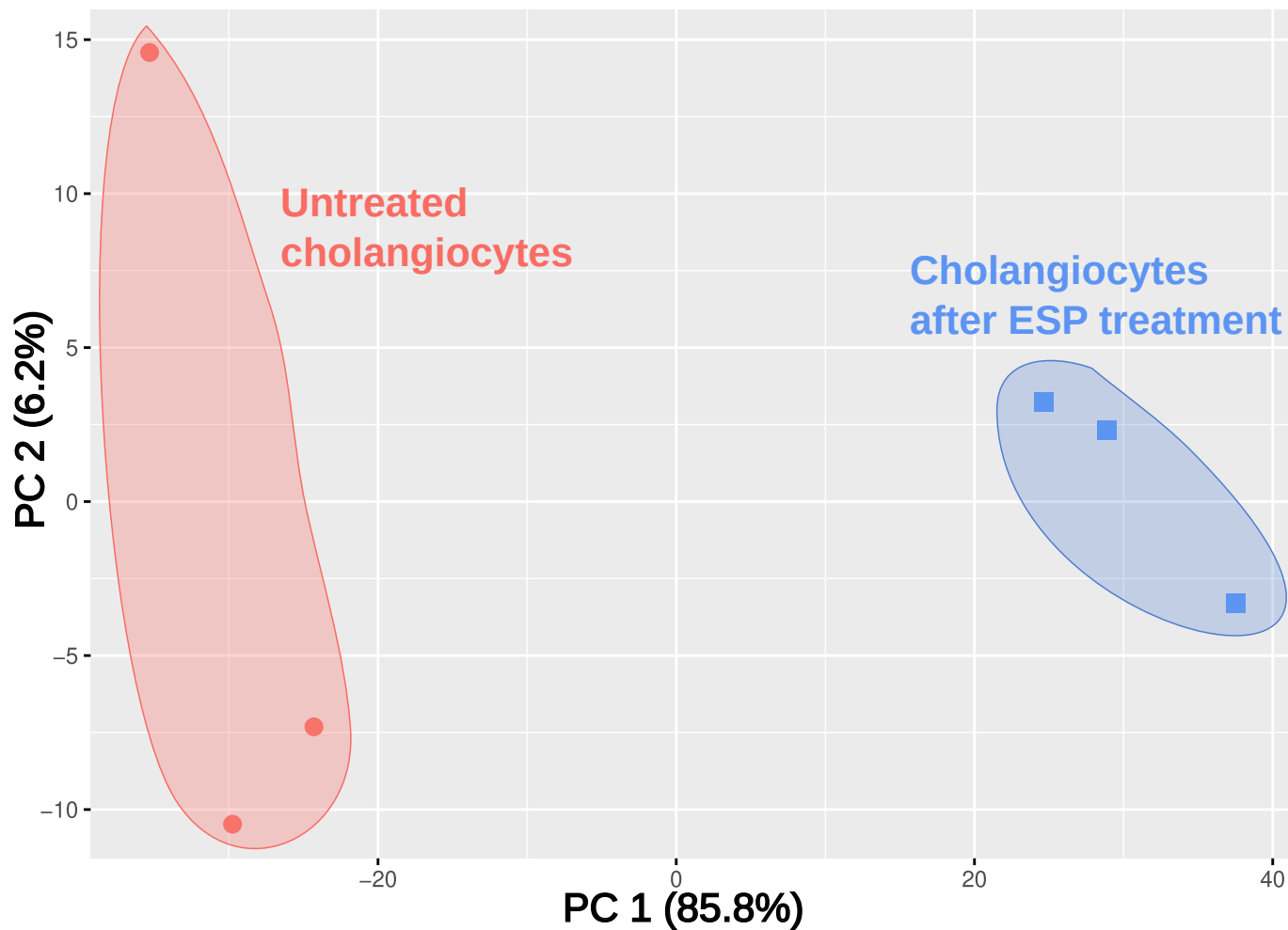

Supplement: Supplementary file 1 [file biomolecules-16-00244-s001.zip › Figure_S2.pdf]

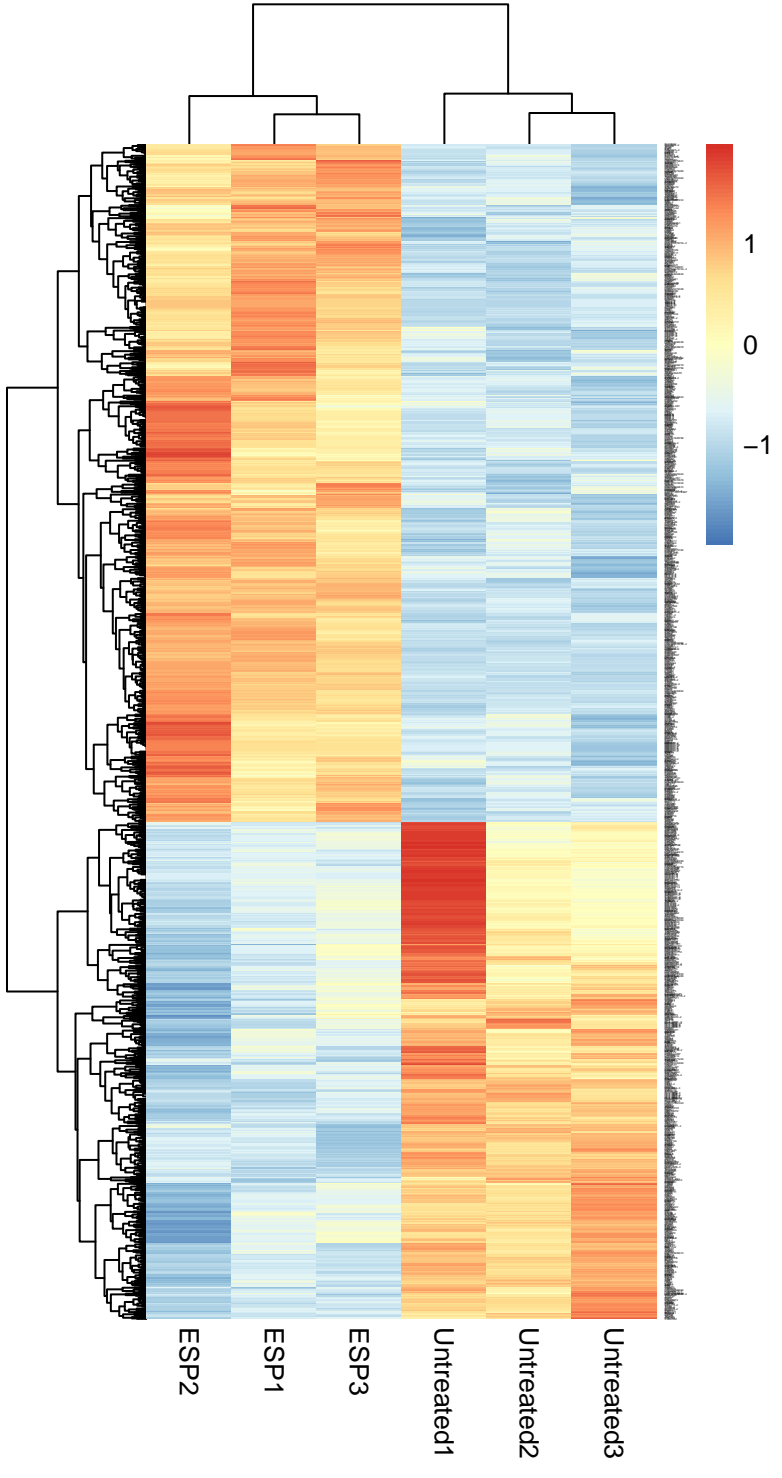

Supplement: Supplementary file 1 [file biomolecules-16-00244-s001.zip › Figure_S3.pdf]
